# Supplementary material for: Synaptic RTP801 contributes to motor-learning dysfunction in Huntington’s disease
Source: Cell Death Dis. 2020 Jul 30;11(7):569. doi: 10.1038/s41419-020-02775-5 (PMC7392897; doi:10.1038/s41419-020-02775-5)
Supplement: Supplementary file 2 — Supplementary Information [file 41419_2020_2775_MOESM2_ESM.docx]

**SUPPLEMENTAL INFORMATION TITLES AND LEGENDS:**

**Figure S1. Synaptosomal enrichment of proteins in the putamen of HD patients.** Putaminal lysates and synaptosomes of 5 HD patients and 5 control individuals were subjected to WB. Membranes were probed against **(A)** RTP801, **(B)** SV2A, **(C)** PSD-95, **(D)** P-Akt (Ser473) and **(E)** P-S6 (Ser235/236) and total Akt as a loading control. Graphs show the densitometric quantification. Data is shown as a mean ± SEM. Data were analyzed with Two-way ANOVA followed by Bonferroni’s multiple comparisons test for post-hoc analyses or Kruskal-Wallis with Dunn’s multiple comparisons (for SV2A) (****P*<0.001 *vs.* CT homogenate) and data of homogenates and synaptosomes were analyzed by Student’s T-test or Mann-Whitney (for SV2A) (**P*<0.05, ***P*<0.01).

**Figure S2. Synaptosomal enrichment of proteins in the cortex of HD patients.** Prefrontal cortex homogenates (H) and synaptosomes (S) of 5 HD patients and 6 control individuals were subjected to WB. Membranes were probed against **(A)** RTP801, **(B)** SV2A, **(C)** PSD-95, **(D)** P-Akt (Ser473) and **(E)** P-S6 (Ser235/236) and total Akt as a loading control. Graphs show the densitometric quantification. Data is shown as a mean ± SEM. Enrichment data was analyzed with Two-way ANOVA followed by Bonferroni’s multiple comparisons test for post-hoc analyses (**P*<0.05, ***P*<0.01, ****P*<0.001 *vs.* CT homogenate; ### *P*<0.001 *vs.* HD homogenate). Data from homogenates was analyzed with Student’s t-test.

**Figure S3. RTP801 is not altered in the synaptic fraction derived from the cortex of HD patients.** **(A, B)** Prefrontal cortex lysates and synaptosomes of 6 HD patients and 6 control individuals were subjected to WB. Membranes were probed against RTP801, P-Akt (Ser473), P-S6 (Ser235/236), PSD-95, SV2A and total Akt as a loading control. Graphs show the densitometric quantification of synaptosomal levels. **(C, D)** Graphs indicate the levels of RTP801, P-Akt (Ser473) and P-S6 (235/236) relative to synaptic markers **(C)** SV2A and **(D)** PSD-95 in the synaptosomes. The results are shown as mean ± SEM. Data were analyzed by Student’s T-test (**P*<0.05, ***P*<0.01).

**Figure S4. Synaptosomal enrichment of proteins in the striatum of HdhQ^7^/Q^111^ mice.** Striatal lysates and synaptosomes of 6 KI and 6 WT animals at 10-months of age were subjected to WB. Membranes were probed against **(A)** RTP801, **(B)** SV2A, **(C)** PSD-95, **(D)** P-Akt (Ser473) and **(E)** P-S6 (Ser235/236) and total Akt as a loading control. Graphs show the densitometric quantification. Data is shown as a mean ± SEM. Data were analyzed with Two-way ANOVA followed by Bonferroni’s multiple comparisons test for post-hoc analyses (***P*<0.01, ****P*<0.001 *vs.* WT homogenate, ##*P*<0.01, ###*P*<0.001 *vs.* KI homogenate) and data of homogenates were analysed by Student’s T-test (**P*<0.05).

**Figure S5. Synaptosomal enrichment of proteins in the striatum of R6/1 mice.** Striatal lysates and synaptosomes of 7 R6/1 and 6 WT animals at 16-weeks of age were subjected to WB. Membranes were probed against **(A)** RTP801, **(B)** SV2A, **(C)** PSD-95, **(D)** P-Akt (Ser473) and **(E)** P-S6 (Ser235/236) and total Akt as a loading control. Graphs show the densitometric quantification. Data in shown as a mean ± SEM. Data were analysed with Two-way ANOVA followed by Bonferroni’s multiple comparisons test for post-hoc analyses (**P*<0.05, ***P*<0.01, ****P*<0.001 *vs.* WT homogenate, #*P*<0.05, ###*P*<0.001 *vs.* R6/1 homogenate) and data of homogenates were analysed by Student’s T-test (****P*<0.001).

**Figure S6. Enrichment of proteins in the striatum of R6/1 after RTP801 knockdown.** Striatal lysates and synaptosomes of WT and R6/1 injected with AAV-shCtr (n=6 WT and n=6 R6/1) or AAV-shRTP801 (n=6 WT and n=7 R6/1) were subjected to WB. Graphs show the densitometric quantification of **(A)** P-mTOR (Ser2448), **(B)** P-S6 (Ser235/236), **(C)** P-Akt (Ser273), **(D)** Rictor, **(E)** PHLPP1, **(F)** GluA1, **(G)** TrkB and **(H)** p75^NTR^. Values are shown as a mean ± SEM. Enrichment data were analyzed with Two-way ANOVA followed by Bonferroni’s multiple comparisons test for *post-hoc* analyses (***P*<0.01, ****P*<0.001 *vs.* WT AAV-shCtr Hom; #*P*<0.05, ##*P*<0.01, ###*P*<0.001 *vs.* R6/1 AAV-shCtr Hom; ; ++*P*<0.01, +++*P*<0.001 *vs.* WT AAV-shRTP801; $*P*<0.05, $$*P*<0.01, $$$*P*<0.001 *vs.* R6/1-shRTP801 Hom).

**Figure S7. Uncropped western blot scans for Figures 2-4 and Figure S3.** Uncropped full scans of western blots from the corresponding cropped western blot panels shown in Figures 2-4 and Figure S3 (red boxes). Molecular weight markers are indicated.

**Figure S8. Uncropped western blot scans for Figures 5-7.** Uncropped full scans of western blots from the corresponding cropped western blot panels shown in Figures 5-7 (red boxes). Molecular weight markers are indicated.
