# Supplementary figures and images for: Synaptic RTP801 contributes to motor-learning dysfunction in Huntington’s disease
Source: Cell Death Dis. 2020 Jul 30;11(7):569. doi: 10.1038/s41419-020-02775-5 (PMC7392897; doi:10.1038/s41419-020-02775-5)

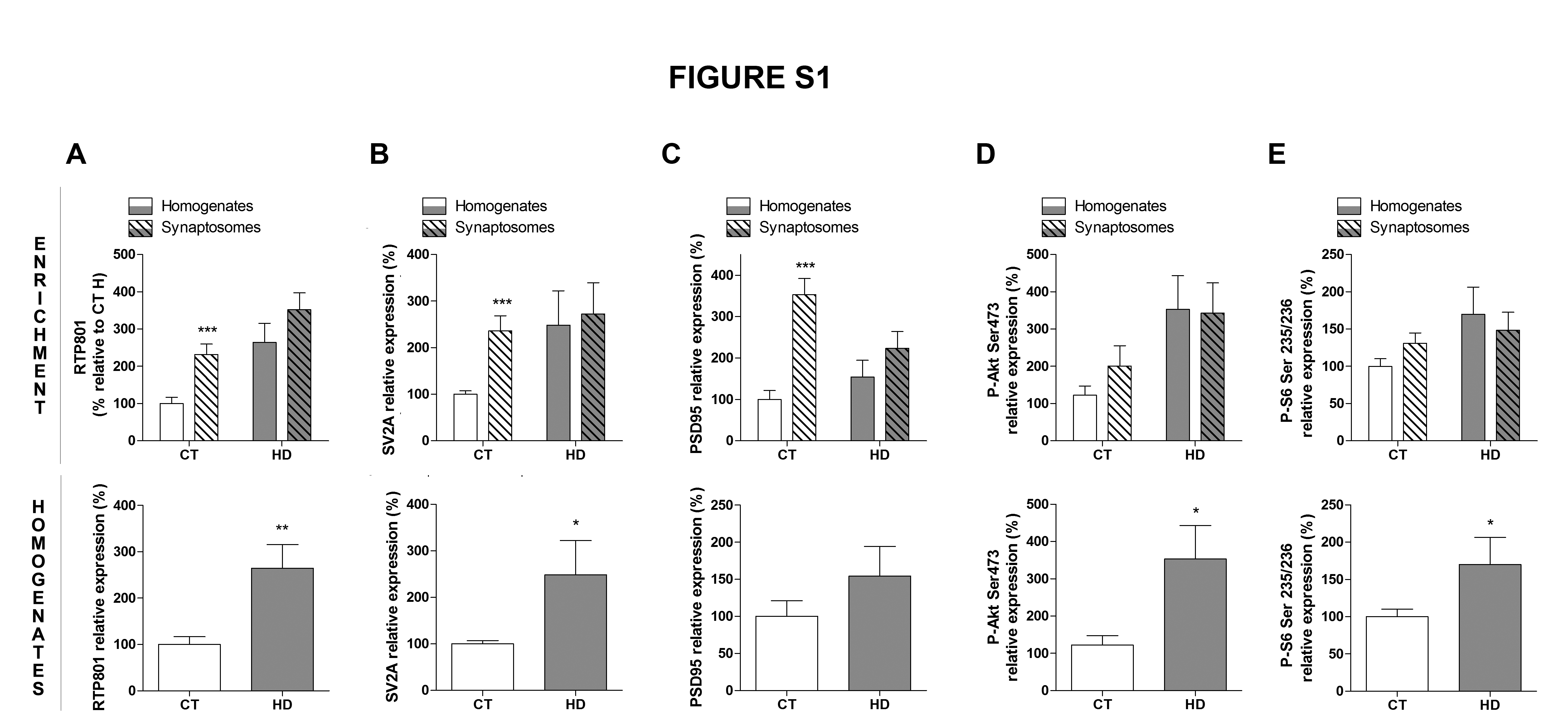

Supplement: Supplementary file 3 — Supplementary FigureS1 [file 41419_2020_2775_MOESM3_ESM.tif]

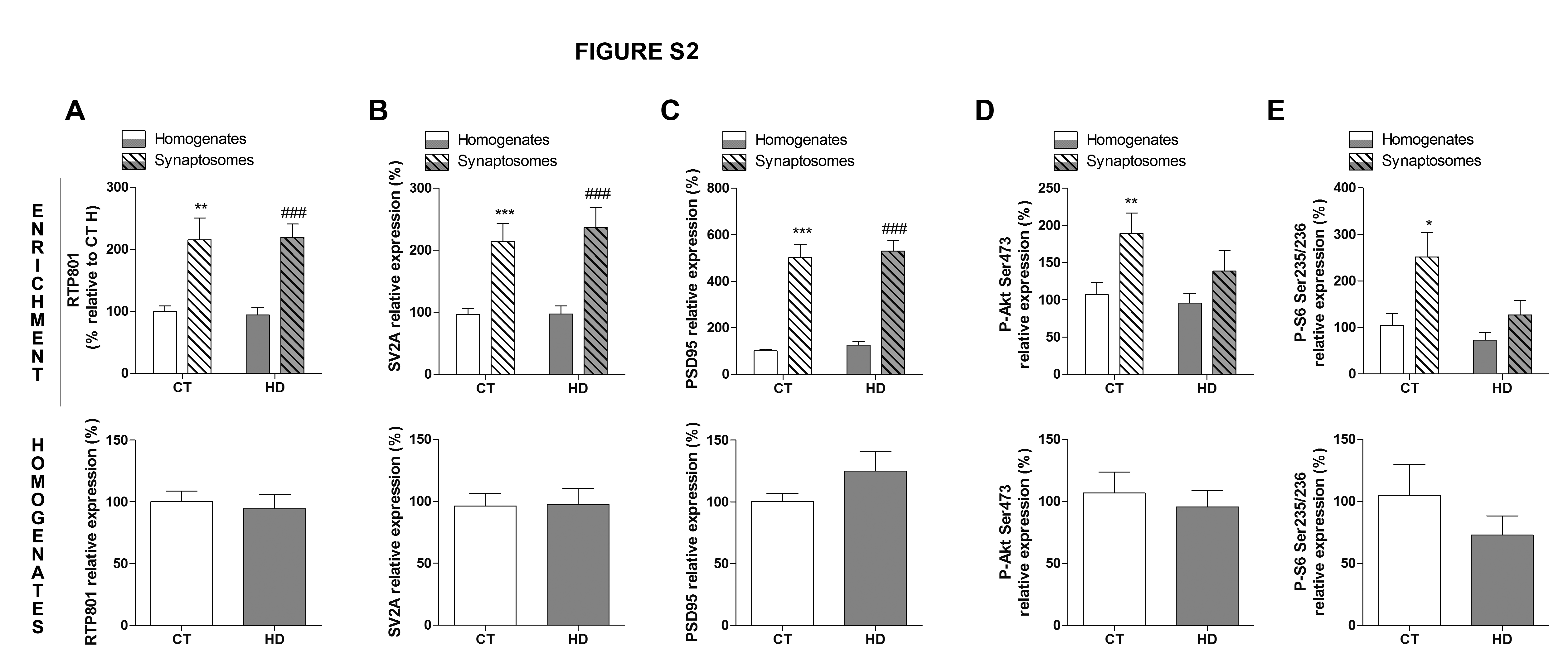

Supplement: Supplementary file 4 — Supplementary FigureS2 [file 41419_2020_2775_MOESM4_ESM.tif]

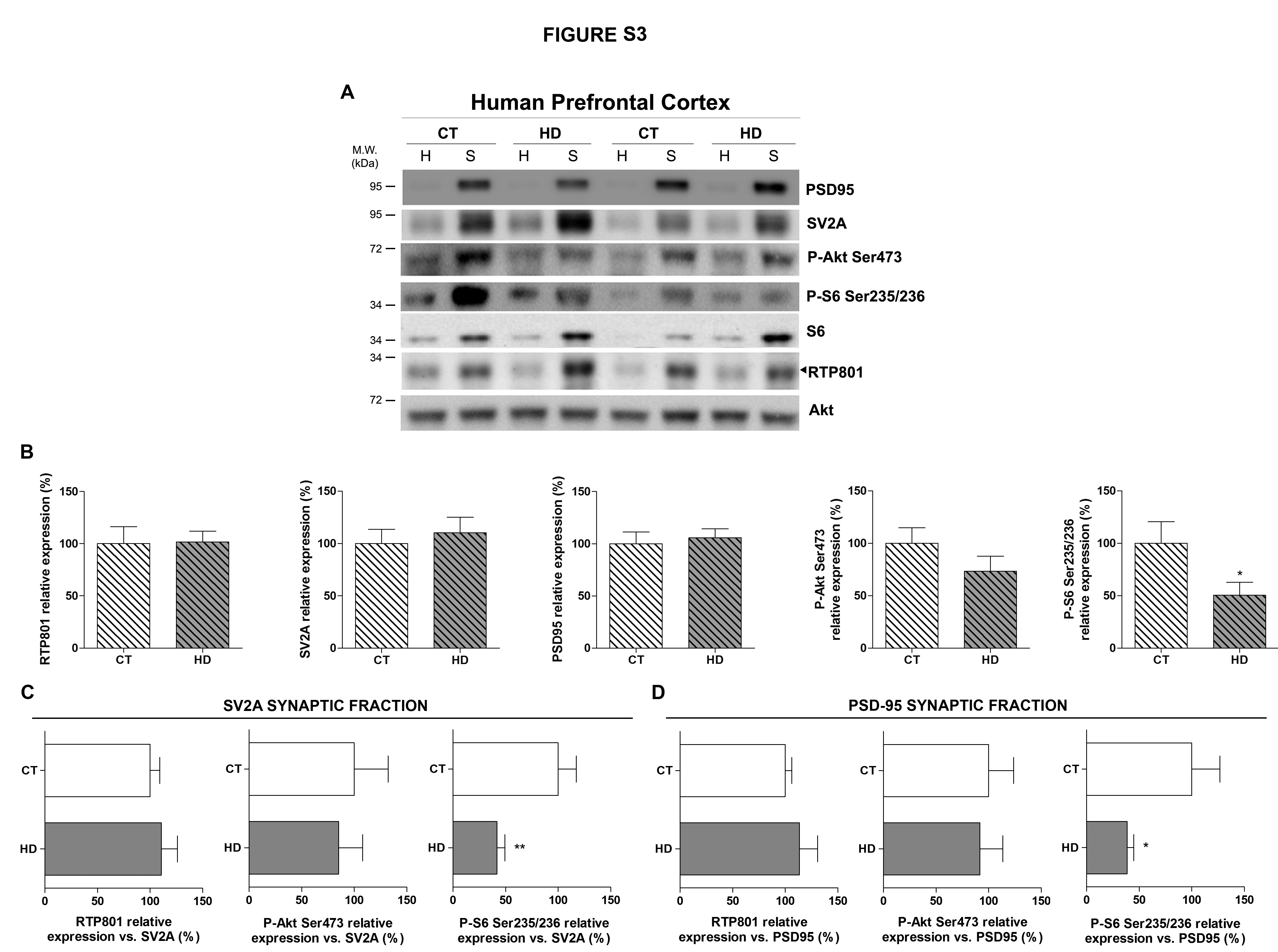

Supplement: Supplementary file 5 — Supplementary FigureS3 [file 41419_2020_2775_MOESM5_ESM.tif]

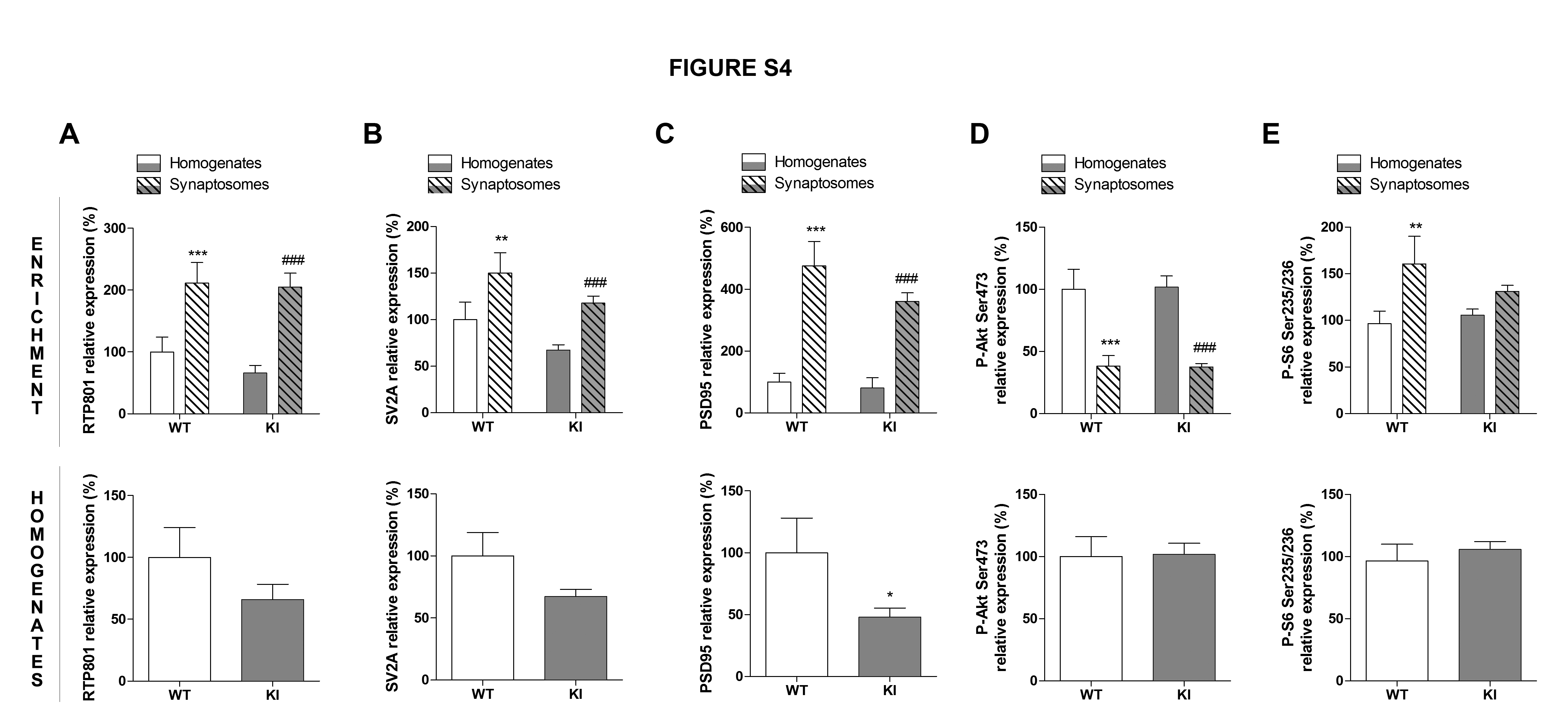

Supplement: Supplementary file 6 — Supplementary FigureS4 [file 41419_2020_2775_MOESM6_ESM.tif]

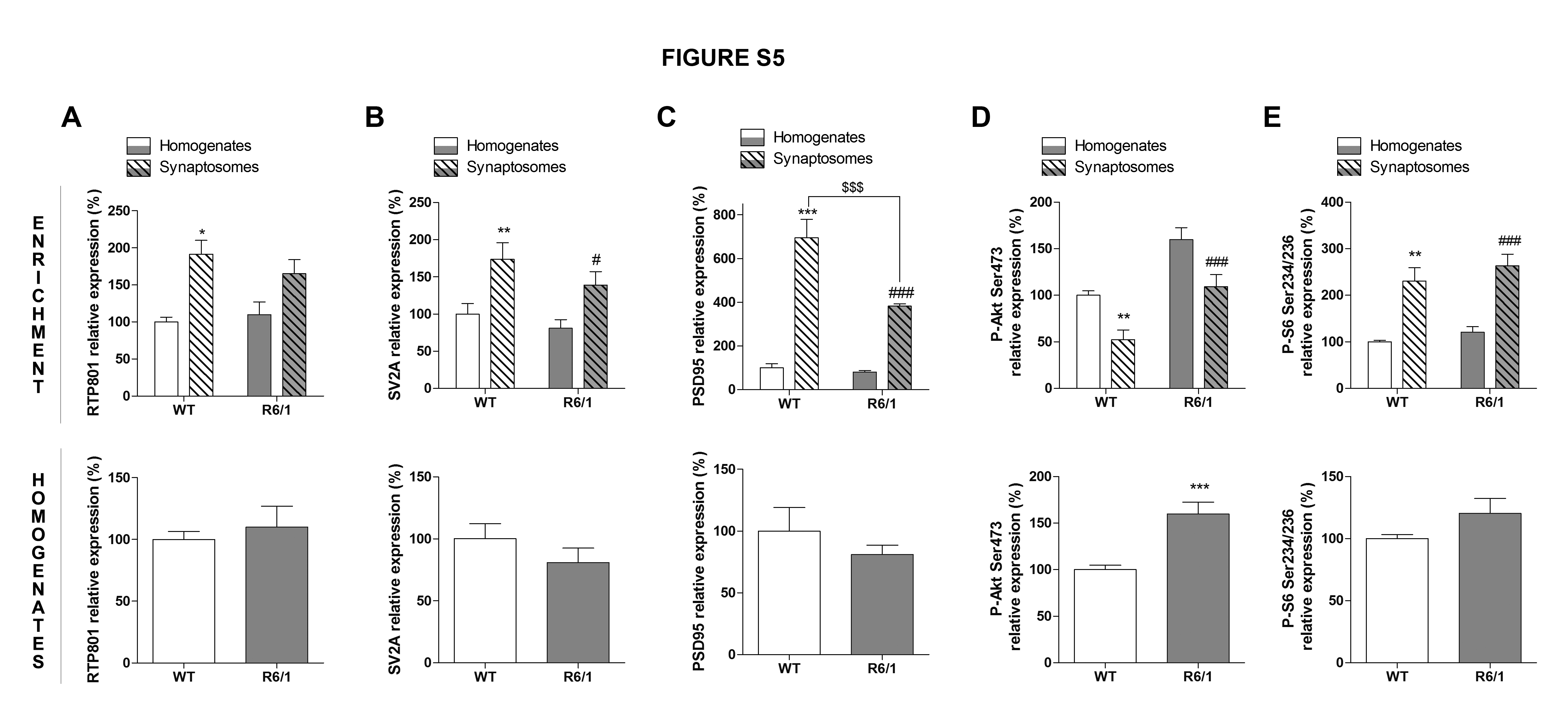

Supplement: Supplementary file 7 — Supplementary FigureS5 [file 41419_2020_2775_MOESM7_ESM.tif]

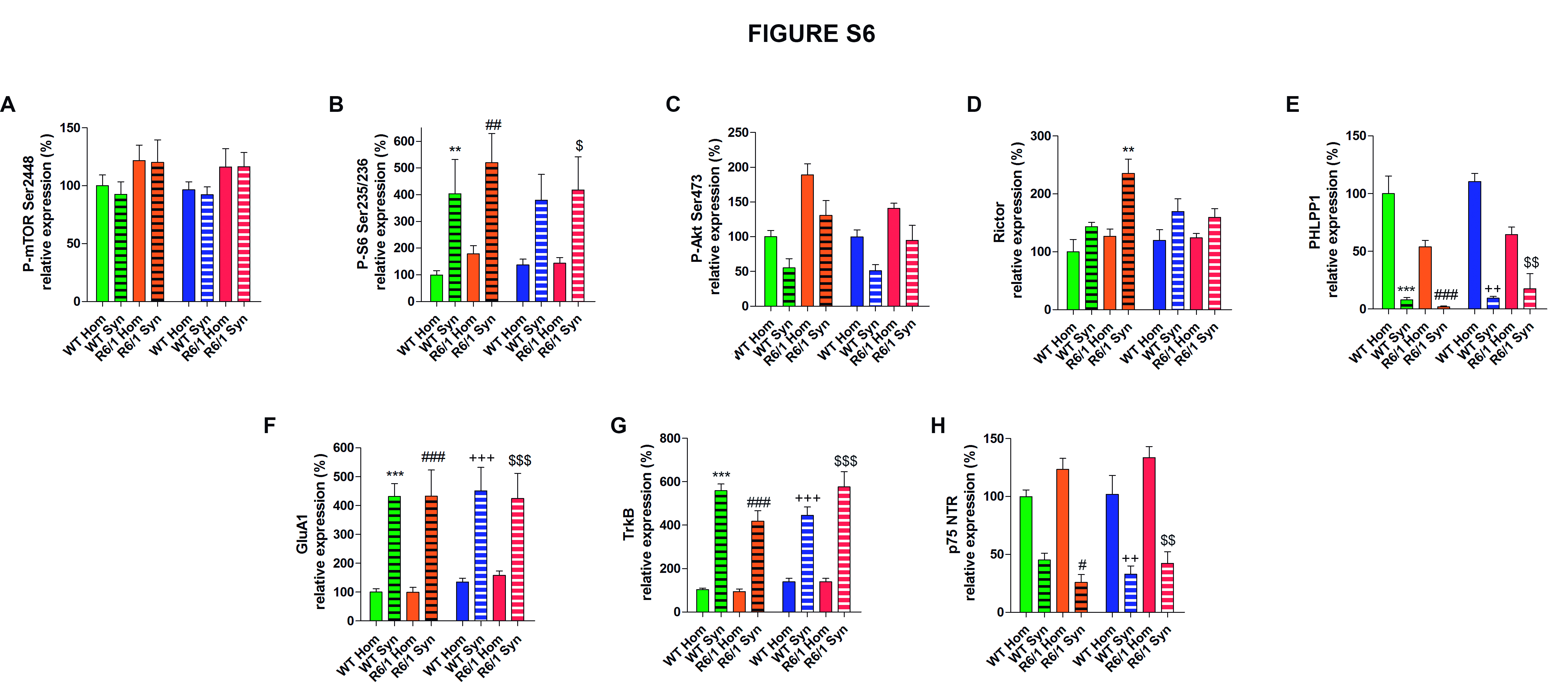

Supplement: Supplementary file 8 — Supplementary FigureS6 [file 41419_2020_2775_MOESM8_ESM.tif]

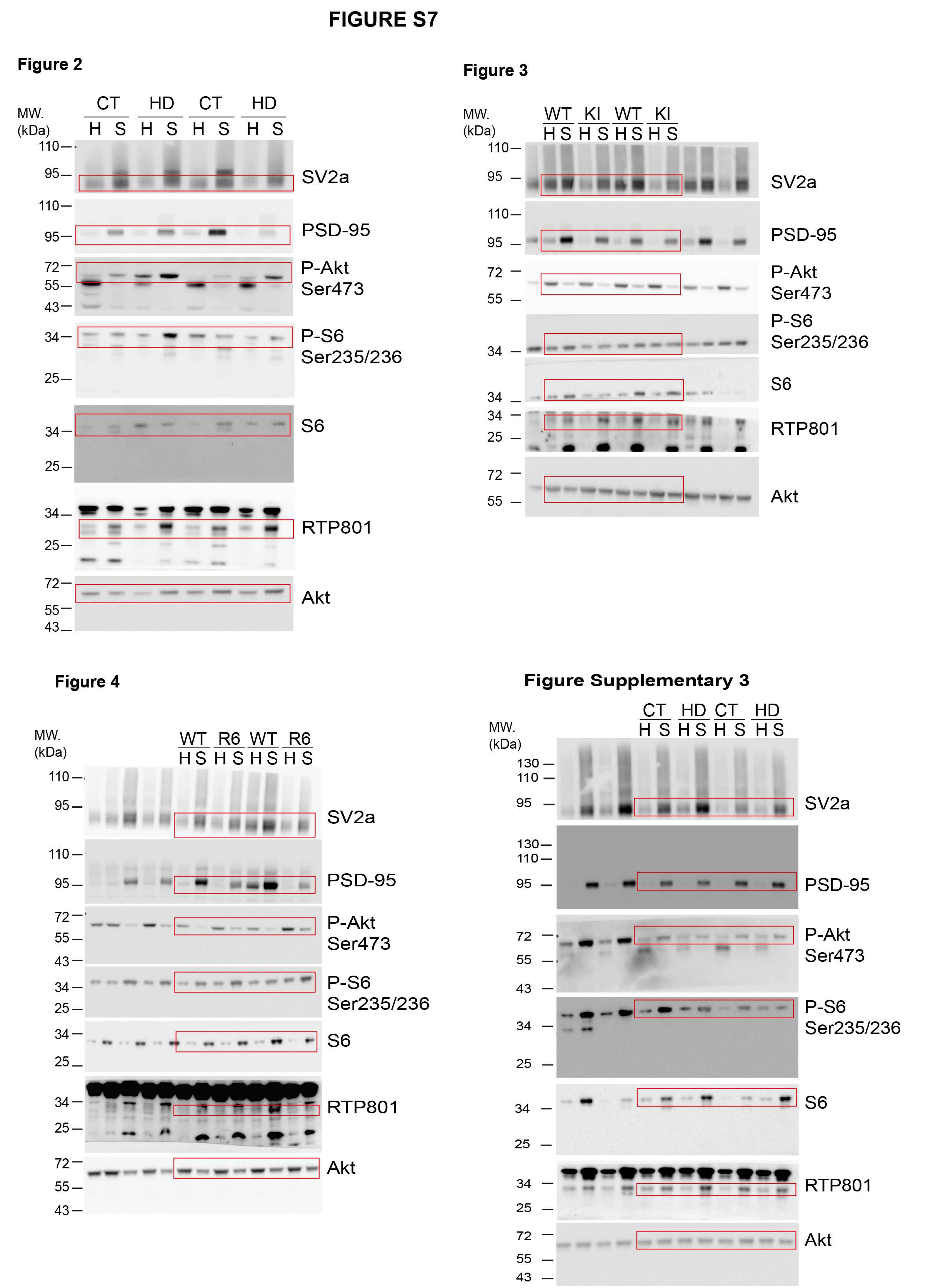

Supplement: Supplementary file 9 — Supplementary FigureS7 [file 41419_2020_2775_MOESM9_ESM.tif]

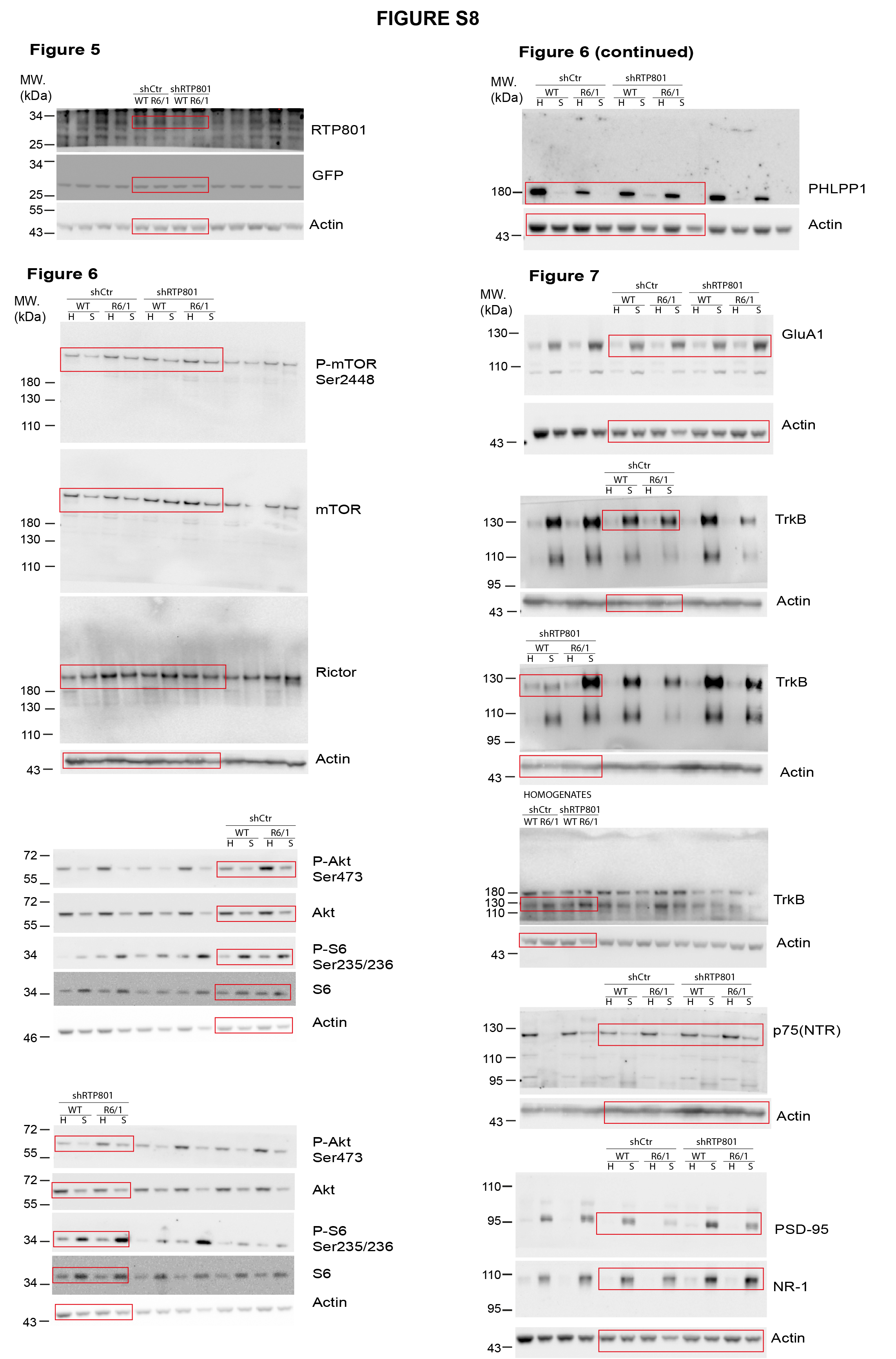

Supplement: Supplementary file 10 — Supplementary FigureS8 [file 41419_2020_2775_MOESM10_ESM.tif]
